# An EEG Study of a Confusing State Induced by Information Insufficiency during Mathematical Problem-Solving and Reasoning

**DOI:** 10.1155/2018/1943565

**Published:** 2018-07-25

**Authors:** Ye Liang, Xiaojian Liu, Lemiao Qiu, Shuyou Zhang

**Affiliations:** State Key Laboratory of Fluid Power Transmission and Control, Zhejiang University, Hangzhou 310027, China

## Abstract

Confusion is a complex cognitive state that is prevalent during learning and problem-solving. The aim of this study is to explore the brain activity reflected by electroencephalography (EEG) during a confusing state induced by two kinds of information insufficiencies during mathematical problem-solving, namely, an explicit situation that clearly lacked information and an implicit situation in which the missing information was hidden in the problem itself, and whether there is an EEG difference between these two situations. Two experimental tasks and three control tasks were created. Short time Fourier transformation (STFT) was used for time-frequency analysis; then the alpha task-related-power (TRP) changes and distributions, which are closely related to cognitive processing, were calculated, and repeated measures ANOVA were performed to find the significant difference between task conditions. The results showed that the alpha power decreased significantly in the regions related to calculation when the participants encountered both explicit and implicit information insufficiency tasks compared to the control tasks, suggesting that confusion can cause more brain activity in the cortical regions related to the tasks that induce confusion. In addition, the implicit information insufficiency task elicited more activity in the parietal and right temporal regions, whereas the explicit information insufficiency task elicited additional activity in the frontal lobe, which revealed that the frontal region is related to the processing of novel or unfamiliar information and the parietal-temporal regions are involved in sustained attention or reorientation during confusing states induced by information insufficiency. In conclusion, this study has preliminarily investigated the EEG characteristics of confusion states, suggests that EEG is a promising methodology to detect confusion, and provides a basis for future studies aiming to achieve automatic recognition of confusing states.

## 1. Introduction

The human working process is a series of problem-solving processes that continually combine existing knowledge in the mind with outside information to achieve desired goals. In terms of the development of technology, the knowledge and information needed for every walk of life is increasing dramatically, especially in the field of complex product design and modeling. Usually, considerable time and energy are necessary for designers to find useful information, though there are already many computer-aided technologies to facilitate such a process. Two main obstacles to achieving an automatic supply of information are “what” and “when”. “What” refers to the finding of information related to specific problems, an aspect that has been thoroughly investigated by researchers in knowledge and information fields. “When” refers to locating the appropriate timepoint at which to provide the related information, which is difficult to achieve with current context-based information service systems, even when the same person is faced with the exact same problems since one's skill and knowledge backgrounds are changing constantly, not to mention the large individual differences. Considering the appropriate time is basically when designers are confused, the feeling that the environment is giving insufficient or contradictory information [1], the most direct and effective approach is to identify people's confusing state, which has seldom been investigated in previous information service studies.

Confusion has been mainly considered an epistemic or knowledge emotion [2, 3], though there still are some theoretical divergences that take it as pure cognitive state [4]. In any case, there is no doubt that emotion and cognition have an inextricable relationship and a continual interaction during problem-solving processes [5, 6]. In the present study, we consider more regarding the cognitive side of confusion because our purpose was to investigate the supplying of information. Before we associate confusion with information supplying, much attention has been drawn to the important role of confusion in learning and education because confusion is prevalent during complex learning, and its impact on learning outcomes can be either beneficial or detrimental [2, 3, 7–10]. In these studies, self-reports and facial expressions are the common methods used to identify a learner's confused state. Both methods have drawbacks that may not reflect an actual confused state or that do not have application prospects; as self-reports are subjective, different learners may have different judgments on confusion, and instant feedback is interruptive, while post-reports reduce timeliness; facial expressions also vary between individuals and can be easily misread. Intuitively, if we can get cognitive information directly from a learner's thoughts, the result should be more accurate. Electroencephalography (EEG) which reflects brain activity may just provide a possible way to do so.

EEG was invented by Hans Berger in 1924 [11]; since then and until now, this method has been widely used in the diagnosis of brain diseases, such as Alzheimer disease [12], epilepsy [13], depression [14, 15], cognitive disorders [16], and brain function research [17–19]. Since the idea that human intentions can be interpreted by EEG activity appeared [20], a great deal of research has focused on the brain-computer interface (BCI), which may help disabled individuals to communicate or control technical devices [21–23]. While clinical applications remain the principal goal of BCI research, the potential of nonmedical applications of BCI technology has drawn increasingly renewed attention [24], especially as wireless and portable EEG devices have been made in recent years [25]. Due to the low reliability and bandwidth of the current BCI systems, a new kind of BCI system, passive BCI, which derives implicit information from arbitrary brain activity without the purpose of voluntary control, is more practical for healthy users [26]. The advantage of passive BCI is that it will not interfere with a user's current work, thus, requiring no need to pay additional attention or effort.

Before the concept of passive BCI was developed, several cognitive states (or mental states) were shown to be reflected in neurophysiological signals, such as workload, fatigue, and stress [27]. The potential use of the recognition of these states has been proposed followed by including monitoring cognitive workload to adjust training difficulty, which can improve learning [28], detecting fatigue during driving in order to prevent potential danger [29], assessing the efficacy of commercial or politics [30], studying the relationship between a designer's mental effort and mental stress, and helping the designers retain proper creativity [31]. Compared to cognitive states such as workload or stress, a confused state is considered slightly more complex. Workload or stress exists during the whole problem-solving process, so their extent, rather than their existence, should be considered. In contrast, confusion may not occur unless one encounters discrepant events, including obstacles to goals, inconsistencies in the information stream, contradictions, anomalous events, and unexpected feedback [2]; thus, not only the intensity of confusion but also when the confusion occurs, what type, and how long it lasts, are issues that need to be studied. Though fully understanding confusion through EEG will take a great deal of continuous work, the present study only partially discusses the confusion induced by the information insufficiency.

When people process well-informed or practiced problems, the brain takes information from outside and long-term memory and loads it into working memory, resulting in specific outcomes; the process is so smooth that it can be done without awareness. If the information needed to solve the problem is insufficient, the process could be interrupted, resulting in the occurrence of confusion. One typical situation occurs when the lack of information is obvious and crucial for the individual to solve the problem, such as the meaning of a foreign language word or the definition of an unknown symbol, requiring the individual to stop the current process and convert to retrieving information if there are possible means available. The individual could clearly be aware of the occurrence of this situation and may experience a “what?” moment where the confusing state began, which would end after receiving the needed information. This is similar to an exceptional problem-solving process, which has been investigated by Anderson, who reported that the cognitive region of the brain is more active during exceptional problem-solving [32]. Another situation may consist of the missing information being hidden in the problem itself, such as a reasoning problem, wherein the individual needs to find the patterns or connections from the information they already have. Although the individual may not feel exceptional because they know something exists that can be revealed by careful deliberation, the confusion still appeared due to the fact that they do not know the right methods unless they actually find it. We describe these two situations as explicit information insufficiency and implicit information insufficiency, respectively. In both situations, effortful cognitive activities are needed in order to resolve the confusion. Meanwhile, if the two confusing states can be distinguished, we can not only provide the information at the right moment, but also provide the information with more accuracy.

To induce confusion, we chose mathematical calculations and reasoning, which are widely used in studies of cognitive processes [32–35]. First, we use an unknown operator calculation to create an explicit information insufficiency situation. For example, consider the simple addition problem, “2 + 3 = ?”. It is easy to solve because people know the number and the meaning of the operator. If we replace the operator with some strange symbol, the calculation becomes unsolvable due to the lack of information, and the individual could become confused when they encounter such problems. To reduce learning effects due to the possibility of participants learning the meaning of a symbol if it appears several times, ten different new operators were created for the experiment, each one representing a unique calculation process and only appearing once. In addition, to prevent the participants from considering these to be nonsense problems and, thus, not even thinking about how to solve them, a new operator equal to the addition operator was also used, and its function was well-known by the participants. This could serve as both a clue and a control group for the unknown operator calculation. For an implicit information insufficiency, inductive reasoning was used as an experimental task. The main purpose of the inductive reasoning was to deduce a number sequence patterns that consisted of the hidden information the participants needed to determine. Inductive reasoning problems can be very difficult to solve if one does not have the right skills; it involves repeated try-verify processes and requires a considerable cognitive demand, resulting in a high workload, which could be the states that usually accompany confusion.

As we considered confusion a cognitive state lasting for a certain time during problem-solving, time-frequency-based approaches were more suitable for our research compared to the study of event-related potentials (ERPs), which reflect a time- and phase-locked response of the brain to a single event [36]. Event-related desynchronization/synchronization (ERD/ERS), representing frequency-specific power changes in the ongoing EEG activity, has been proven as a useful method to reflect the activation level of cortical areas involved in processing cognitive information [37]. Within the frequency range of EEG, a great deal of previous studies have demonstrated that a decrease in alpha band power is functionally related to active cognitive processing [38–42]. Thus, we hypothesized that cognitive load and activities would increase when the participants were solving the experimental tasks, which would be indicated by a desynchronization of the EEG alpha band. Furthermore, confusion was believed to trigger deep thought, and individuals who were confused would be more engaged and vigilant [2, 5]. Thus, tasks inducing confusion are expected to cause a different level of the alpha ERD, and the difference between explicit and implicit situations can be reflected by the different activities of various brain regions.

## 2. Materials and Methods

**Figure 1:**
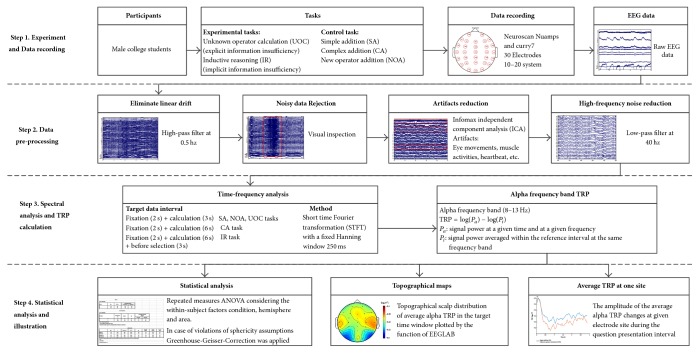
Framework for analyzing the confusing states induced by information insufficiency.

Figure 1 provides a framework for analyzing the confusing states, which is composed of four major steps.

**Step 1 (experiment and data recording).**

EEG data of all the participants were recorded by 30 electrodes amplifier while they were performing the two experiment tasks and the three control tasks.

**Step 2 (data preprocessing).**

High- and low-pass filter, visual inspection, and infomax independent component analysis algorithm (ICA) were applied on the raw EEG data recorded in Step 1 to reduce noise and artifacts.

**Step 3 (spectral analysis and TRP calculation).**

The power of target data interval was obtained by using short time Fourier transformation (STFT) to transform the data into time-frequency domain; then the alpha band task-related power (TRP) was calculated by the formula in the diagram and as the input data for analyzing EEG characteristics of confusion states.

**Step 4 (statistical analysis and illustration).**

The amplitude of alpha TRP changes at all electrode sites during the question presentation interval was averaged across all participants and topographical scalp distributions of average alpha TRP in the target time window were plotted by the function of EEGLAB. Repeated measures ANOVA were performed to find the significant difference between task conditions.

### 2.1. Participants

Twenty-three right-handed male students participated in the experiment as paid volunteers. All participants had normal or corrected-to-normal vision and reported no medical or psychiatric illness. All participants gave written informed consent prior to the experiment. This study was approved by the Ethics Committee of the Zhejiang University.

### 2.2. Experimental Tasks

Five types of mathematical problems were used as experimental tasks (Figure 1, Step 1). The reason why we chose mathematical calculation and reasoning was that there is no background knowledge needed for participants to solve them, and it is easy to control the difficulty or solvable of the tasks. The details and examples of the tasks are presented in Table 1. The experiment consisted of 60 problems total, with each type having 10 problems except for the inductive reasoning (IR) task, which had 20. Each problem had 2 options, and the participants needed to choose the one they believed to be the answer.

The simple addition (SA) task was considered a normal task with low cognitive demand that would not lead to confusion. Participants could easily solve this kind of problem without hesitation. We chose 2-digit numbers with 1 carry to ensure the participants did perform a mental calculation process since memory retrieval is predominantly used by participants to solve problems if they are too easy (e.g., 2 + 5 = 7) [36, 43].

The complex addition (CA) task was more difficult than simple addition, in that it could take participants more time to solve it and required a greater cognitive workload; however, this task is still well-defined and is not confusing. This kind of task was considered a controlled condition to IR task.

The new operator addition (NOA) task contained the same problems as the simple addition, only with a new operator “$”, the function of which was equal to “+”. This type of problem was devised as an intermediate level control task of simple addition and the unknown operator calculation. Participants were told the meaning of “$” and had practiced before the formal experiment. The new addition operator represented newly learned information that the participants needed to solve the problem; thus, though simpler, the participants would respond to the task in a similar way as we do to a normal working condition. The task may have required slightly more cognitive demand than simple addition due to the information being stored in short-term memory and the lack of practice. However, we hypothesized that the brain activity in response to this task would be similar to that in response to simple addition because each involves basically the same calculation process. Meanwhile, if there was an EEG difference between SA and UOC tasks, we wanted to know if it was elicited by the confusing state or the additional process induced by the unfamiliar operator.

The unknown operator calculation (UOC) task was a calculation task with ten distinct operators. The meanings behind these operators each represented different calculation methods. Participants were not informed either of the meaning or the existence of this kind of problem which would confuse the participants. Each operator only appeared once in order to avoid participants being able to guess the meaning from the options. Details and examples of the ten operators have been presented in Table 2.

Inductive reasoning (IR) has been widely used in intelligence and aptitude tests and has also been used in studies of problem-solving [44, 45]. It usually appears as a series of numbers, wherein participants need to find the pattern hidden in the series and reason the next number or the missing one between them. An example of an easy one would be the following: “1, 3, 9, ? ”. Clearly this example is a geometric progression and the answer would be “27”. The difficult problem required several transformations and could take the participants a considerable amount of cognitive demand. We did not choose easy ones because they were not challenging to the participants, as it was too easy to figure out the pattern. If the problem was too hard to solve, participants would lose faith and start using guessing strategies, deviating from confusing state. Therefore, we choose 20 problems; among them, 10 problems were relatively easier than the other ten and could be solved using the same strategies as those of the practice problems before the formal experiment. Even if they were to do so, the participants may not be able to solve all of these ten problems, but at least some success should have given them faith to do more thinking rather than guessing. Additionally, we wanted to make a comparison between situations in which the participants think they got the right answer and the ones in which they doubt it. There was no feedback stage after any of the problems, because we were focused on the confusing state during the problem-solving process and did not want participants to learn from the right answers.

### 2.3. Experimental Procedure

**Figure 2:**
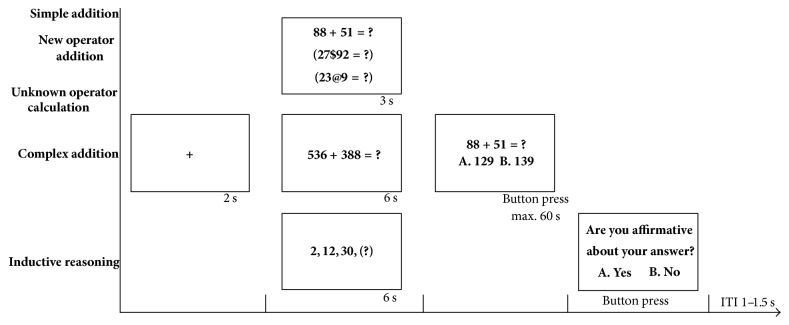
Procedure of the experimental task presentation. Every trial began with a 2-sec fixation cross in the middle of the screen, which was followed by the task questions that were presented for 3 sec for the simple addition (SA), new operator addition (NOA), and unknown operator calculation (UOC) tasks and for 6 sec for the complex addition (CA) and inductive reasoning (IR) tasks. The options then appeared under the question and were presented for a maximum of 60 sec until the participants pressed the button. A survey appeared after the IR tasks only. Trials were separated by an intertrial interval (ITI) of ~1–1.5 s randomly.

The whole experiment had two parts, namely, a practice stage and an experimental stage. In the practice stage, participants could practice all the task types, except for the UOC task, with the same procedures as the experimental stage. Once the participants confirmed that they fully understood the types of the tasks and the experimental procedure, they could choose to start the formal experiment. In the experimental stage, every problem started with a 2 s fixation presented at the center of a display. Then, problems appeared without options in pseudorandom order. For the SA, NOA, and UOC problems, there was 3 s before the options would show up and 6 s for the other two problems. The purpose of not letting the problems and options appear together was to avoid the participants guessing the answer based on the options at the beginning, which left them with a period of free time to think and calculate. This procedure also reduced the participants' temptation to press the button when they observed the options, which would produce motor cortex activity. It took the participants at least 3 s to solve the SA task and 6 s to solve the CA task in our pretest. In addition, it certainly took more time for the other three types of tasks, so this is the time interval for analysis. Only two options were used to minimize the hand and neck activity of the participants that was needed to look for and confirm the key. There was no demand for participants to quickly respond in order to avoid additional mental pressure, and there was a 60 s time limit after the onset of the options only for process control purpose for cases when the participants spent too much time on one problem. In case the participants selected the right answer by guessing or chose the wrong answer due to miscalculation, a survey question appeared only after the IR problems. Participants needed to answer whether they were certain they got the right answer. If participants failed to make a selection within the 60 s or if the problem was one of the other three types, no survey appeared and there would be an intertrial interval (ITI) of ~1–1.5 s randomly. Then, the next problem's fixation began. To avoid fatigue, there was a rest period after every fifteen problems, and the participants could rest as long as they wanted to. Details of the procedure are shown in Figure 2.

Participants were seated comfortably at approximately 80 cm from a 23-in. (16:9) computer screen, with a keypad in hand, where key “1” and key “3” were used to make the choice. They were instructed to try to avoid body movements while performing the tasks.

### 2.4. EEG Recording and Analysis

The electrical signals in the brain were recorded using an elastic cap with electrodes at 30 scalp sites, according to the 10–20 system (NuAmps and curry7; Neuroscan, Australia) (Figure 1, Step 1). Reference electrodes were placed on the right and left mastoids, and a ground electrode was applied to the forehead. Additionally, vertical eye movements were recorded with electrodes that were placed above and below the left eye, and horizontal eye movements were measured by electrodes placed on the outside rims of both eyes. The impedance of all the electrodes was maintained below 5 kΩ. The EEG and EOG signals were continuously sampled at 1 kHz for offline analysis.

The recorded EEG data were preprocessed using EEGLAB [46] and MATLAB. The data were high-pass filtered at 0.5 Hz to eliminate linear drift of the DC amplifier. Then, strange periods with large amounts of noise were rejected by visual inspection. Then, the data were entered into the infomax independent component analysis algorithm [46–48]. The independent components related to eye movements, muscle activities, heartbeat, or other clear artifacts were removed, and an additional 40 Hz low-pass filter was then performed (Figure 1, Step 2).

Short time Fourier transformation (STFT) with a fixed Hanning window (250 ms) was used for the time-frequency analysis [42]. Brain activity during the performance of the tasks was quantified by means of task-related power (TRP) changes in the EEG data, which are widely used as indicators of ERD/ERS in cognitive brain research [37, 49, 50]. TRP at an electrode was calculated by subtracting the log-transformed power of reference intervals from the log-transformed power of the activation intervals according to the following formula: TRP = log⁡(*P*_*a*_) − log⁡(*P*_*i*_), where *P*_*a*_ is the signal power averaged at a given time interval and at a given frequency and *P*_*i*_ is the signal power averaged within the reference interval at the same frequency band (Figure 1, Step 3). A negative TRP value corresponds to decreases in power (ERD) from the reference to the task interval, whereas increases in power (ERS) are expressed as positive value. A 1-s interval during the fixation period (−50 to −1050 ms before problem onset) was used as a reference for the TRP calculations. As we mentioned in the introduction, the alpha frequency band power is closely related to cognitive processing, and the ERD/ERS of the alpha band can indicate the activation level of the given cortex area. Therefore, we chose the alpha band (8–13 Hz) for further analysis. Considering that the participants were understanding the superficial meaning of problems at the beginning of the problem onset period, a 1-s sample of the EEG data was taken after 1000 ms of the SA, NOA, and UOC problem onset and a 2-s sample of the data after 1500 ms of the CA and IR problem onset for further analysis. To investigate the effect of difficulty, another 1-s sample of the data was extracted after 3000 ms of the CA task onset, in the middle of the calculation process, to compare with the SA task. In addition, we were also interested in the brain activity before the button press of the IR problems. Three 1-s EEG data segments before the button presses of two conditions (Certain-Uncertain) were analyzed. Trials in which artifact-free data could not meet the above time requirement were excluded from further analyses. Topographical scalp distributions of average alpha TRP in the above time window were plotted by the function of EEGLAB and the amplitude of alpha TRP changes at all electrode sites during the question presentation interval of the five tasks were averaged across all participants.

For statistical analyses, electrode positions were chosen and topographically aggregated as follows: prefrontal (PF) left (FP1), frontal (F) left (F3, F7), frontocentral (FC) left (FC3, FT7), centroparietal (CP) left (C3, CP3), temporal (T) left (T3, TP7), parietotemporal (PT) left (P3, T5), and occipital (O) left (O1), as well as analogous locations for the right hemisphere [49]. The midline electrodes (FZ, FCZ, CZ CPZ, PZ, and OZ) were not included in the analyses, as we also wanted to explore hemispheric differences. Repeated measures ANOVA were used for statistical analyses. In case of violations of sphericity assumptions, the Greenhouse-Geisser-Correction was applied (Figure 1, Step 4).

## 3. Results

### 3.1. Behavioral Results

Two participants whose EEG data had too much noise were excluded from further analyses. Additionally, two participants who made a choice in less than 3 s for almost all the IR problems were excluded from the IR problem analysis. The amount of correctly answered problems was varied from 48 to 54, with an average of 50.9, as shown in Table 3.

### 3.2. EEG Results

Paired comparisons were made for the five task types as follows: SA-UOC, SA-NOA, NOA-UOC, CA-IR, SA-CA, and Certain-Uncertain. TRP changes in the alpha band were analyzed for these pairs using repeated measures ANOVA considering the within-subject factors' condition (SA-UOC, SA-NOA, NOA-UOC, CA-IR, SA-CA, and Certain-Uncertain), hemisphere (left-right), and area (prefrontal (PF), frontal (F), frontocentral (FC), centroparietal (CP), temporal (T), parietotemporal (PT), and occipital (O)).

**Figure 3:**
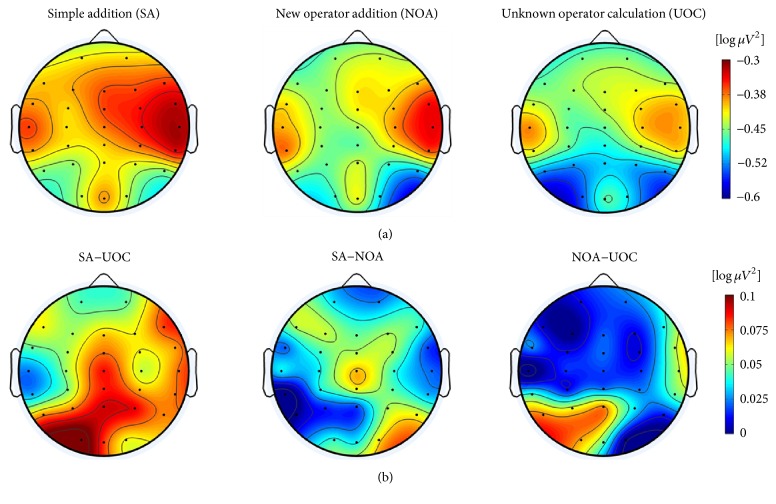
Topographical maps of the SA, NOA, and UOC tasks. Topographical maps of the distribution of alpha TRP changes in the 1-2 s time window of the SA, NOA, and UOC tasks are presented in the first row, respectively, and the second row displays the differences between each two of the tasks.

**Figure 4:**
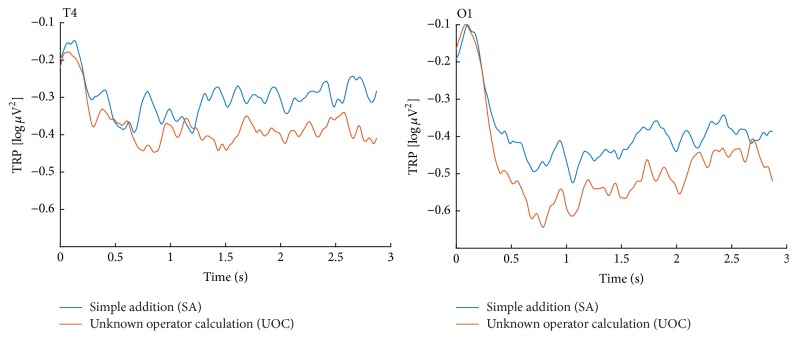
Alpha TRP changes in the SA and UOC tasks. The amplitude of the alpha TRP changes at the T4 and O1 electrode sites during the question presentation interval.

**Figure 5:**
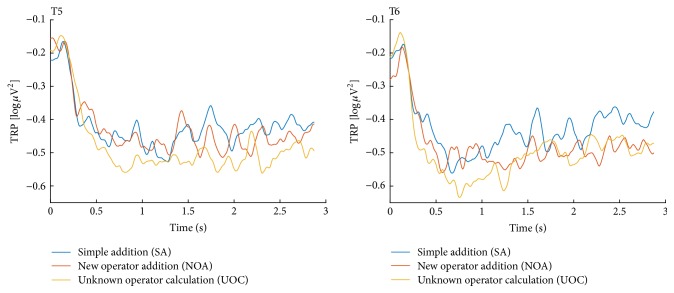
Alpha TRP changes in the SA, NOA, and UOC tasks. The amplitude of the alpha TRP changes at the T5 and T6 electrode sites during the question presentation interval.

**Figure 6:**
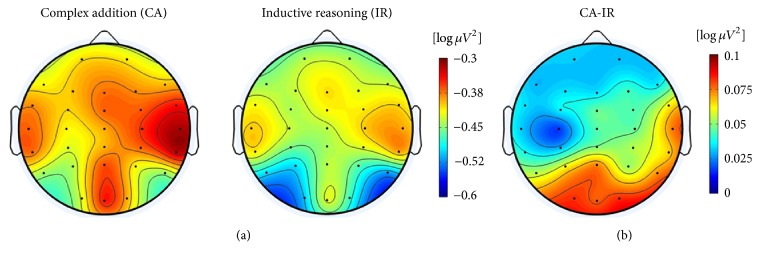
Topographical maps of the CA and IR tasks. Topographical maps of the distribution of alpha TRP changes in the 1.5–3.5 s time window of the CA and IR tasks are presented in (a), and the difference between them is presented in (b).

**Figure 7:**
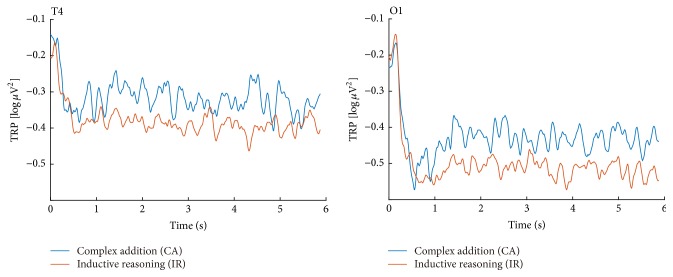
Alpha TRP changes of CA and IR tasks. The amplitude of alpha TRP changes at the T4 and O1 electrode sites during the question presented interval.

For the SA-UOC contrast, the 2 × 2 × 7 ANOVA suggested that the main effects of condition, hemisphere, and area were significant (*F*(1,20) = 6.53, *p* = 0.019, partial-*η*^2^ = 0.246; *F*(1,20) = 5.04, *p* = 0.036, partial-*η*^2^ = 0.201; *F*(6,120) = 5.53, *p* = 0.016, partial-*η*^2^ = 0.217, respectively), which indicated that the UOC problems showed more task-related alpha decreases, and the alpha power was significantly lower in the frontal and occipital areas of the left hemisphere, as well as the temporal area of right hemisphere (as Figures 3 and 4 show). However, the interaction between these factors did not reach statistical significance. Similar results also appeared with the CA-IR contrast (as Figures 6 and 7 show) (*F*(1,20) = 6.17, *p* = 0.022, partial-*η*^2^ = 0.236; *F*(1,20) = 6.04, *p* = 0.023, partial-*η*^2^ = 0.232; *F*(6,120) = 3.68, *p* = 0.038, partial-*η*^2^ = 0.155, respectively). Considering the SA-NOA and NOA-UOC contrasts, only the effects of area were statistically significant (*F*(6,120) = 5.09, *p* = 0.019, partial-*η*^2^ = 0.203; *F*(6,120) = 6.12, *p* = 0.009, partial-*η*^2^ = 0.234, respectively), but there was a tendency towards a main effect hemisphere (*F*(1,20) = 3.85, *p* = 0.064, partial-*η*^2^ = 0.162; *F*(1,20) = 3.31, *p* = 0.084, partial-*η*^2^ = 0.142, respectively). In addition, the SA-NOA contrast showed a tendency towards a main effect condition, whereas the NOA-UOC contrast did not (*F*(1,20) = 3.80, *p* = 0.066, partial-*η*^2^ = 0.159; *F*(1,20) = 1.22, *p* = 0.283, partial-*η*^2^ = 0.057, respectively). This effect suggested that the alpha power tended to lower for the NOA task than for the SA task. The TRP changes were more similar to the UOC task. Although they did not reach statistical significance, the TRP changes of the NOA tasks at the left parietotemporal, left occipital, and right temporal areas were lower than those of the UOC tasks and more similar to the SA tasks (as Figures 3 and 5 shows). Meanwhile, only the effects of hemisphere were statistically significant within the SA and CA tasks (*F*(1,20) = 0.102, *p* = 0.752, partial-*η*^2^ = 0.005; *F*(1,20) = 5.288, *p* = 0.32, partial-*η*^2^ = 0.209, *F*(6,120) = 1.848, *p* = 0.172, partial-*η*^2^ = 0.85; main effects of condition, hemisphere, area respectively), revealing that they had the same level alpha TRP changes.

**Figure 8:**
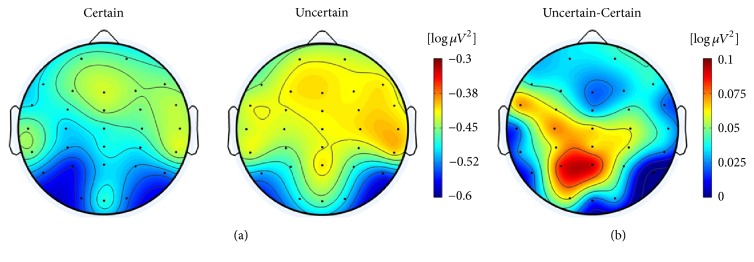
Topographical maps of the IR tasks. Topographical maps of the distribution of alpha TRP changes in the penultimate sec time window before selection under the “certain” and “uncertain” conditions are presented in (a), and the difference between them is presented in (b).

For the certain-uncertain situation, a significant effect of area (*F*(6,108) = 5.368  *p* = 0.01, partial-*η*^2^ = 0.23) and a tendency towards a significant effect of condition (*F*(1,18) = 3.969, *p* = 0.062, partial-*η*^2^ = 0.181) were observed only in the second 1-s data sample before selection. In addition, the alpha power in the uncertain situation was significantly lower in the left frontocentral and centroparietal regions than in the certain situation (as Figure 8 shows).

## 4. Discussion

In the present study, we focused on the task-related alpha power change evoked by solving different mathematic problems to explore the confusing state. The results showed that task-related power decreased for five tasks at all electrodes. A higher level of alpha desynchronization was observed both in the UOC and IR tasks, while almost the same lower level was observed for the SA and CA tasks. The alpha TRP changes of the NOA task had similar features to the SA and UOC tasks, though in different brain regions. Additionally, the alpha power increased slightly before selection if the participants were uncertain about their answer.

In the selected data interval, the alpha power decreased from the reference interval over all cortical areas in all five tasks, as we hypothesized (see Figures 3 and 6). This phenomenon was also observed in previous studies that involved mental arithmetic [36, 51]. It is assumed in current neurophysiological models that the alpha desynchronization is related to cortical activation and reflects actual cognitive information processes [37, 38, 52]. Thus, this suggests that even the simplest calculation is a complex process that involves long-term memory, short-term memory, and calculation strategies and that correlates with different cortical regions [33]. In addition, all five tasks were bottom-up processes that needed sustained access to the information in the stimulus, which is also believed to result in lower task-related alpha power [49, 53, 54]. In addition, the left hemisphere has shown greater alpha ERD than the right hemisphere, in general, and while our experimental tasks were mainly about the calculation process, the results were in line with previous fMRI studies that reported that several left brain cortical regions were involved in calculations [32, 55, 56].

The UOC task evoked more alpha power desynchronization than the SA task, especially at the bilateral frontal, parietal, left occipital, and right temporal areas (see Figures 3 and 4). The UOC task is a typical situation of explicit information insufficiency. Unlike the SA task, which the participants could smoothly process, the operator symbols in the UOC task are seldom used in normal calculations, resulting in inconsistencies in the information stream, and participants could not solve this kind of problem unless they knew the meaning of the operator. Even when this crucial information was missing, the cognitive activities were intensified instead of weakened despite the unsolvable nature of the problems, a finding that was supported by the higher level alpha desynchronization. An explanation of this may be that the confusion induced by the unknown operators triggered more related brain activity, such as that involved in connecting to each participant's own knowledge structure to guess or reason the possible meaning of the operators. This was in line with studies suggesting that confusion can trigger deep thought and that participants intended to resolve the confusion by engaging in deeper cognitive activities [2, 5, 7].

For the NOA task, contrary to our hypothesis, a near significant condition effect on the alpha TRP changes was observed compared to the SA task. The left frontal and right occipital areas showed lower task-related alpha power during NOA task processing, and a similar tendency at the left frontal and right parietal areas (Figure 3). Moreover, no significant condition effect on the alpha TRP was observed between the NOA task and the UOC task, which also suggested that the brain activities of the NOA task were generally more similar to the UOC task. Noting that the NOA task had the same calculation difficulty as the SA task, the only difference was the new addition operator that the participants had learned before the formal experiment, which should not have induced confusion. One possible reason for this could be that the new addition operator was newly learned information, still loaded in short-term memory and easily mixed up with other ten unknown operators due to lack of practice. Once participants encountered the NOA task, the new addition operator activated more brain region, such as the bilateral frontal area which was previously reported to be related to working memory [56]. Meanwhile, the frontal and parietal regions are considered metacognitive regions that can maintain activation after exceptional problem-solving [32]. Therefore, even if the participants realized the meaning of the symbol and made the same calculation as the SA task, the related metacognitive regions were still activated. However, the left parietotemporal, left occipital, and right temporal regions had similar alpha TRP changes in the SA task (as Figures 3 and 4 shows). This suggests that the activation in this region might involve actual calculation processes that overlapped with the metacognitive processing, which is in line with previous mental calculation studies [56–59]. Another possible reason is that the participants might have been more concentrated and engaged when facing a new addition operator since the frontal and parietal regions are also believed to be associated with controlled attention processes [60, 61].

Considering the IR task, a situation of implicit information insufficiency, the hidden information was the pattern of the number series, which demanded that the participants try and test repeatedly until they find the answer. Due to the lack of well-defined solution, unless the participants got the final answer, every attempt was a subprocess containing the following: making assumptions, testing calculations, and detecting impasses, which potentially triggered confusion [2, 5]. The brain activity difference between the CA and IR tasks was quite similar to the SA and UOC tasks. A Higher alpha ERD appeared at the bilateral parietotemporal, occipital, right centroparietal, and temporal areas during IR task processing. As mentioned above, the bilateral parietal lobe is considered to play a key role in the calculation process, and the left parietal regions also contribute to the function of verbal coding of numbers, and, thus, has greater precision in numerical coding [61]. Furthermore, the activation of the right parietal and temporal regions has been reported to be related to the reorientation of attention function, and the IR task typically needs a different approach to be solved [32]. This interpretation could also account for the different activity of these regions in the SA, NOA, and UOC tasks. Except for the similarity, the UOC task elicited more frontal brain activity than the SA task, while no significant difference was observed between the IR and CA tasks. This is possible because an explicit information insufficiency is usually accompanied by some novel or unknown features, such as the unknown operator in the UOC task. However, both the IR and CA tasks did not have such features involved, thus suggesting that manipulations of novel information that is held in working memory require more cognitive demand than familiar information does.

Another interesting finding was that although a study has reported that more complex calculation processes produce higher alpha ERD [36], the present results reflected no significant difference between the alpha TRP changes in the SA and CA tasks, and the topographical maps of those two tasks were almost identical. There is no doubt that CA task processing should involve more cognitive demand than the SA task. This may have been due to the small number of participants and trials; however, it has been suggested that more cognitive activity is required when processing unknown or unfamiliar information and confusion can cause more brain activity in cortical regions related to tasks that induce confusion.

As for the comparison between the certain and uncertain situations before selection in the IR task, the alpha power increased slightly, especially at the left frontocentral and left centroparietal areas in the uncertain situation, while the level remained the same in the certain situation, which was contrary to our expectation. It should be noted that when the participants were uncertain about their answer it meant that they felt that the problem was too difficult to solve and, consequently, chose to use the guessing strategy to select the answer. An interpretation of this phenomenon could be that when participants failed to find the pattern after several attempts, their mental state transformed from confusion to frustration or boredom, thus stopping the calculation process, which resulted in reduced cognitive activity at related brain regions. These dynamics of cognitive emotion that have frequently appeared at challenging learning process may also appear in complex problem-solving [7, 9].

The present study that utilized alpha TRP changes revealed that the confusion induced by either an explicit or implicit information insufficiency resulted in more brain activity in cortical regions related to the ongoing tasks, providing EEG evidence that there are tight links between cognitive processes and confusion [7]. However, the stimuli used in the experiment were mathematic problems in which the information involved is limited to numbers, operators, or calculation strategies. While confusion is a complex cognitive state that can appear in a variety of situations, it is still unknown whether this preliminary result can be extended to other situations of information insufficiency; thus, future studies are required to investigate this issue.

## 5. Conclusion

Our results suggested that, during the processing of five types of tasks, the alpha power decreased at all electrodes, especially in the parietooccipital region. In addition, a higher level alpha desynchronization was observed during the UOC and IR task-solving processes than during the well-handled tasks, such as the SA and CA tasks, at related brain regions, which indicated that more cognitive effort was needed during the confusing state. Furthermore, the frontal region showed more activity when processing novel or unfamiliar information, and activities in the parietal and temporal regions reflected sustained attention or reorientation that was elicited by the information insufficiency; all these regions play an important role in the recognition of a confusing state.

## Figures and Tables

**Table 1:** Experimental tasks.

Task type	Feature	Example
Simple addition (SA)	2-digit numbers with 1 carry	88 + 51 = ?
Complex addition (CA)	3-digit numbers with 2 carries	536 + 388 = ?
New operator addition (NOA)	2-digit numbers with 1 carry	27$92 = ?
Unknown operator calculation (UOC)	10 distinct operators	23@9 = ?
Inductive reasoning (IR)	5-number sequences	4, 4, 3, 2, (?)

**Table 2:** Unknown operators.

Operator	Meaning	example
︾	Minus	74 ︾ 25 = 49
@	Multiplication	23@9 = 207
*※*	Division	832 *※* 16 = 52
*◎*	Decrease continuous multiplication	5 *◎* 3 = 5*∗*4*∗*3 = 60
*︘*	Remainder	123 *︘* 40 = 3
Θ	Rounding	98Θ20 = 4
Ψ	Power	7Ψ2 = 49
*＊*	Increase continuous multiplication	2*＊*5 = 2*∗*3*∗*4*∗*5 = 120
#	Continuous addition	1 # 10 = 1 + 2 + ⋯ + 9 + 10 = 55
︺	Rooting	64︺2 = 8

**Table 3:** Behavioral results.

Task type	Mean correct	Mean RT (ms)	certain correct
SA	9.90	1400.51	/
NOA	9.86	1751.30	/
CA	10	2382.01	/
UOC	7.57	10959.44	/
IR	13.71	23057.81	10.4
